# Vascular Abnormalities in Peripapillary and Macular Regions of Behcet's Uveitis Patients Evaluated by Optical Coherence Tomography Angiography

**DOI:** 10.3389/fmed.2021.727151

**Published:** 2021-09-16

**Authors:** Chun Yan, Fan Li, Min Hou, Xiaoyuan Ye, Lishi Su, Yixin Hu, Jiawen Luo, Wei Chi

**Affiliations:** ^1^State Key Laboratory of Ophthalmology, Zhongshan Ophthalmic Center, Sun Yat-sen University, Guangzhou, China; ^2^Department of Ophthalmology, Luoyang Central Hospital, Zhengzhou University, Luoyang, China

**Keywords:** Behcet's disease, retinal microstructure, optical coherence tomography angiography (OCTA), Behcet's uveitis, vasculitis

## Abstract

**Purpose:** To investigate the involvement of peripapillary zone vascular abnormalities in Behcet's uveitis (BU) and associated visual dysfunction. We evaluated the retinal and choroidal microvascular features in both macular and peripapillary areas of BU patients to identify vascular abnormalities contributing to reduced best-corrected visual acuity (BCVA) using optical coherence tomography angiography (OCTA).

**Methods:** A prospective, observational study was conducted in 24 eyes of 13 patients with BU and 24 eyes of 15 healthy participants as controls. They received a standard eye examination and were recorded by OCTA measurements of macular and peripapillary areas. The vascular densities of superficial capillary plexus (SCP) and deep capillary plexus (DCP), choroidal flow area, radial peripapillary capillary network (RPCN) density, foveal avascular zone (FAZ) area and perimeter, full retinal thickness (FRT), and peripapillary retinal nerve fiber layer thickness (pRNFLT) were measured.Correlations among microvascular, structural, and functional changes were assessed.

**Results:** Our findings uncovered that the vascular density was significantly reduced in the peripapillary zone of BU eyes compared to healthy eyes, especially in the inferior subfield of the RPCN. The vascular densities of SCP and DCP quadrants within the macular zone had no significant difference between BU and control groups except for DCP density of the nasal parafoveal quadrant. Both FAZ area and perimeter were greater but without statistical significance in the BU group. Compared to healthy eyes, the choriocapillaris flow area was smaller while the FRT and pRNFLT were greater in the BU group. Notably, there was a significant correlation between the reduction in RPCN vascular density and decreased BCVA in BU patients.

**Conclusion:** Based on OCTA, vascular changes associated with BU are more prominent in the peripapillary zone than those in the macular zone. The vascular density of the RPCN could serve as a sensitive indicator to monitoring BU pathogenic progression and treatment response using a non-invasively method of OCTA.

## Introduction

Behcet's disease (BD) is a multisystem immune-mediated disorder manifested by recurrent uveitis (Behcet's uveitis, BU), oral or genital ulcers, and skin lesions (1–3). BD has a worldwide distribution (4), and over two-thirds of BD cases present with bilateral recurrent uveitis and stubborn retinal microvasculitis (5–7). BU is one of the most stubborn and refractory uveitis entity in the worldwide, leading to severe vision loss.

Fluorescein angiography (FA) can detect both the leaky and the occlusive nature of retinal vasculitis in BU (6). However, the invasiveness (8) and time consuming restrict the application of FA in long-term follow-ups of BU patients. Moreover, FA cannot visualize multiple layers of the retinal vasculature separately or resolve radial peripapillary and deep capillary networks clearly (9). In comparison, OCTA is a non-invasive and transformative imaging technique that can quantify the retinal and disc microcirculation rapidly and accurately down to the capillary level (10). Furthermore, OCTA can reveal the retinal vascular structure layer by layer, which provides micromesh information about structural damage (11). Combined with structural optical coherence tomography (12), OCTA enables a more comprehensive understanding of vascular abnormalities in some eye diseases, including diabetic retinopathy (13), glaucoma (14, 15), age-related macular degeneration (16), retinal vein obstruction (17, 18), and Vogt-Koyanagi-Harada disease (19).

Previous OCTA studies have revealed macular vascular abnormalities in BU patients, including perifoveal capillary disorders (20, 21), reduced vascular density (22). However, no reports have been made on performing peripapillary analyses of patients with BU using OCTA, so the associations between OCTA-based peripapillary zone abnormalities and visual dysfunction remain unclear. We aim to comprehensively analyze vascular and structural changes in the macular and peripapillary zones of BU patients using OCTA and to examine the associations with best-corrected visual acuity (BCVA) deficits.

In the present study, we disclosed a significant decrease in vascular density within the peripapillary region of BU patients compared to healthy subjects. Further, BCVA was positively correlated with vascular density in the peripapillary region, suggesting that the density of radial peripapillary capillaries may be a novel indicator for disease progression and prognosis among BU patients.

## Materials and Methods

### Study Design and Ethical Approval

This cross-sectional comparative study was approved by the Ethics Committee of Zhongshan Ophthalmic Center (Guangzhou, China 2019KYPJ127) and conducted in compliance with the principles of the Declaration of Helsinki. Informed consent was obtained from each participant prior to enrollment.

### Study Subjects

Inclusion criteria were patients fulfilling the diagnostic criteria for BU developed by the International Study Group for Behcet's Disease (ISGBD) (23)and presenting with posterior uveitis or panuveitis characterized by retinal vasculitis and manifestations of optic nerve and/or macular inflammation. Investigational work-ups (e.g., serological testing for syphilis, chest x-ray, tuberculin skin test, and anterior chamber tap for viral polymerase chain reaction) were performed in selected cases to rule out conditions mimicking BU. Patients with other retinal and/or optic nerve diseases or who underwent intraocular surgery other than for uncomplicated cataract were excluded. Age- and sex-matched healthy individuals were recruited as a control group. Poor quality OCTA images resulting from media opacities, eye movements, or incorrect auto-segmentation were also excluded from further analysis.

All patients received detailed ophthalmic examinations, including the assessment of BCVA, slit-lamp biomicroscopy, intraocular pressure assessment, and dilated fundus examination. Color fundus photography and FA were also performed, followed by OCTA scan (Optovue Inc., Fremont, CA, USA).

### Layer and Sector Segmentation in OCTA

**Figure 1:**
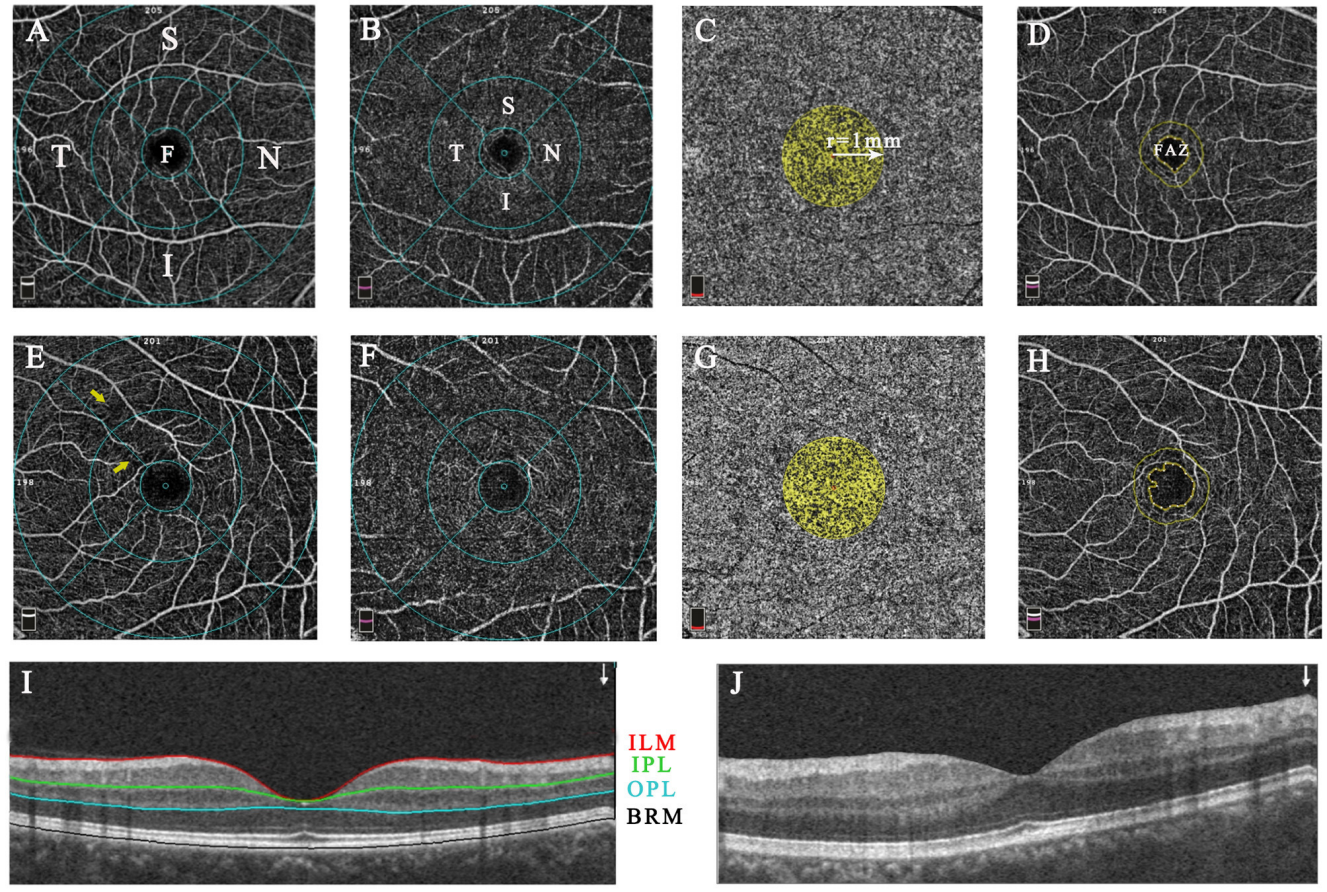
The representative macular image diagram of layer and sector segmentation and vascular parameter measurements in healthy **(A–D,I)** and Behcet's uveitis subjects **(E–H,J)**. The images were acquired within a 6.0 × 6.0-mm macular cube scan centered on the fovea in 4 default en-face slabs: the SCP (ILM to IPL; **A,E**), the DCP (IPL to OPL; **B,F**), the CC (BRM to BRM+30 μm; **C,G**), and the retina slab (ILM to OPL; **D,H**). B-scan image shows the place of segmentation **(I,J)**. The SCP and DCP layers were overlaid by the ETDRS grid comprised of 3 concentric rings of 1, 3, and 6-mm diameter; The fovea, parafovea, and perifovea were defined as the inner circle, medial annulus, and outer annulus, respectively. The vascular density was automatically measured in different subfields of SCP and DCP **(A,B,E,F)**. The CC flow area was calculated within a 1-mm radius circle centered at the fovea **(C,G)**. The FAZ area and perimeter were quantified basing on the retina slab, and automated FAZ boundary detection was performed by the AngioVue software **(D,H)**. Capillary arcade disruptions with rarefaction of vessels (yellow arrow) were present in the temporal and superior sectors of the BU patient. F, fovea; S, superior; T, temporal; I, inferior; N, nasal; SCP, superficial capillary plexus; ILM, inner limiting membrane; IPL, inner plexiform layer; OPL, outer plexiform layer; CC, choriocapillaris; BRM, Bruch's membrane; DCP, deep capillary plexus; ETDRS, the Early Treatment Diabetic Retinopathy Study; FAZ, foveal avascular zone.

**Figure 2:**
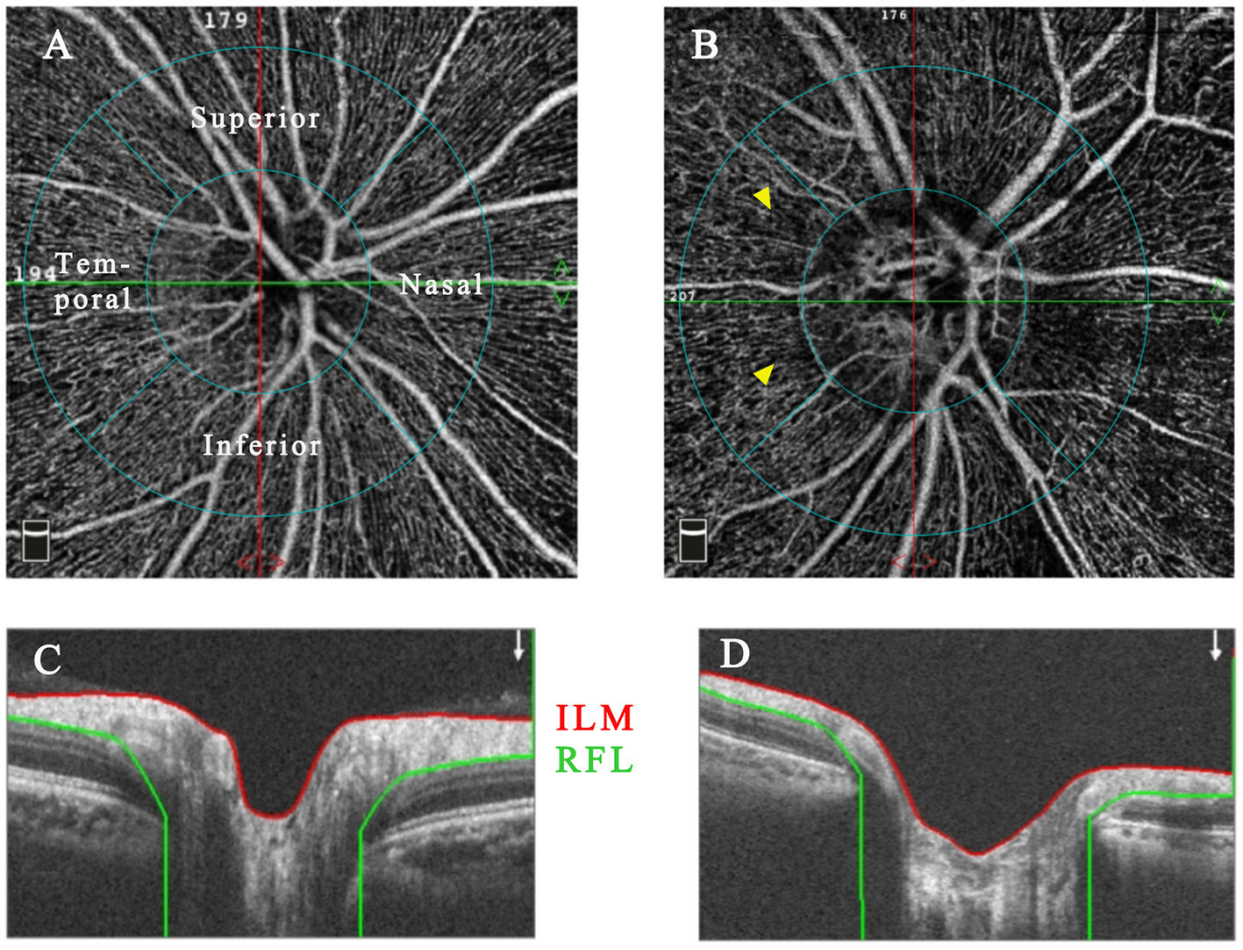
The representative RPCN OCTA images with accompanying B-scan images of healthy **(A,C)** and Behcet's uveitis eyes **(B,D)**. RPCN images were acquired between the ILM and NFL within a 4.5 × 4.5-mm cube scan centered on the optic disc. The peripapillary region was an annular area with 1-mm-width, and the inner concentric ring was defined by the disc boundary. Grayish areas of capillary non-perfusion/hypoperfusion were observed in OCTA images of a Behcet's uveitis patient (yellow arrowhead). OCTA, optical coherence tomography angiography; RPCN, radial peripapillary capillary network; NFL, nerve fiber layer.

En-face OCTA slabs of the superficial capillary plexus (SCP), deep capillary plexus (DCP), choriocapillaris, and retinal layers within the macular zone, and the radial peripapillary capillary network (RPCN) within the peripapillary zone were automatically segmented by the built-in software AngioVue (Software Version 2017.1, RTVue XR Avanti, AngioVue). The software automatically divided the peripapillary and macular (parafovea and perifovea) regions into four sectors (temporal, superior, nasal, and inferior) for quadrant analysis and two hemispheres (superior-hemi and inferior-hemi) defined by a horizontal line drawn through the disc or foveal center. The diagrams of layer and sector segmentation within macular and peripapillary regions are displayed in Figures 1, 2.

### Macular Parameter Measurements

A 6.0 × 6.0-mm macular cube OCTA scan centered on the fovea was acquired to obtain the following parameters: (1) vascular densities of SCP and DCP (defined as % areas occupied) in separate sectors of the macular region; (2) vascular flow area in the choriocapillaris, calculated within a 1-mm radius circle centered on the fovea; (3) foveal avascular zone (FAZ) parameters, including FAZ area and perimeter, quantified in the retina slab extending from the inner limiting membrane (ILM) to the outer plexiform layer (OPL); (4) full retinal thickness (FRT), which was set to incorporate the slab from the ILM to the retinal pigment epithelium (RPE) and was generated from different subfields of the macular region.

### Peripapillary Parameter Measurements

Each studied eye underwent a 4.5 × 4.5-mm cube OCTA scan centered on the optic disc. The parameters measured in the peripapillary zone included (1) radial peripapillary capillary network (RPCN) vascular densities (exclusive of large vessels) and (2) peripapillary retinal nerve fiber layer thickness (pRNFLT) calculated from the ILM to the nerve fiber layer (NFL) across different peripapillary sectors.

### Statistical Analyses

Statistical analyses were performed using SPSS version 24 (SPSS Inc., Chicago, IL). All quantitative data are presented as mean ± standard deviation (SD). Conformity to the normal distribution was tested using the Shapiro–Wilk procedure. Independent samples *t*-test was used to compare normally distributed data and Mann-Whitney U test to compare non-normally distributed data. Pearson χ^2^ test was used to compare categorical variables. Pearson's correlation analyses were performed among log MAR BCVA, peripapillary parameters, and macular parameters. A *P* value < 0.05 (two-tailed) was considered statistically significant for all tests.

## Results

### Demographic Summary

Thirteen BU patients and 15 healthy participants were enrolled in this study at the Zhongshan Ophthalmic Center, Sun Yat-Sen University, China, between July 2019 and October 2019. Demographic data and clinical details are summarized in Table 1. There were no significant differences in mean age or sex ratio between groups. In the BU group, two eyes were excluded from analysis due to poor-quality images. Among included patients (mean age 32.9 ± 15.2 years, range 14–56 years), ocular involvement was bilateral in 12 cases (92.3%) and unilateral in one (7.7%). The log MAR BCVA ranged from 0 to 1.70 and the mean was 0.87 ± 0.62.

**Table 1:** Characteristics of BU Patients and Normal Controls.

**Characteristics**	**BU**	**Normal**	***P value***
Subjects (eyes)	13 (24)	15 (24)	
Gender, male (ratio)	7 (53.8%)	5 (33.3%)	0.149
Age, years (range)	32.9 ± 15.2 (14–56)	38.5 ± 12.4 (19–58)	0.257
Laterality			
Unilateral (ratio)	12 (92.3%)	NA	
Bilateral (ratio)	1 (7.7%)	NA	
log MAR BCVA	0.87 ± 0.62	NA	

*BU, Behcet's uveitis; log MAR, logarithm of the minimum angle resolution; BCVA, best-corrected visual acuity*.

### Macular Microcirculation Parameters

The microcirculation analyses of the macular region for BU and healthy eyes are summarized in Table 2. The flow area in the choriocapillaris was dramatically lower in BU eyes than controls (*P* = 0.009). The vascular densities of both SCP and DCP within the macular region did not differ significantly between BU and healthy eyes, except in the nasal parafoveal quadrant of the DCP (*P* = 0.048, Supplementary Table 1), whereas qualitative vascular abnormalities were presented in the SCP of BU patients (Figure 1). Both FAZ area and perimeter were identifiably greater in the BU group, but without significance compared to healthy controls.

**Table 2:** Comparison of FAZ Parameters, Flow Area and Vascular Densities between BU and Normal Eyes.

**Variables**	**BU**	**Normal**	***P value***
Flow area-Choriocapillaris (mm^2^, at 1 mm radius)	1.91 ± 0.24	2.37 ± 0.13	0.007^**^
FAZ area (mm^2^)	0.99 ± 2.07	0.38 ± 0.25	0.629
FAZ perimeter (mm)	3.52 ± 4.04	2.19 ± 0.30	0.959
**Vascular density-SCP (%)**			
Whole image	45.81 ± 5.12	45.07 ± 4.07	0.618
Fovea	23.26 ± 15.58	17.81 ± 6.57	0.133
Parafovea	42.41 ± 6.02	44.34 ± 5.77	0.301
Perifovea	47.75 ± 6.55	46.04 ± 4.15	0.326
**Vascular density-DCP (%)**			
Whole image	44.41 ± 7.41	44.16 ± 7.48	0.912
Fovea	33.26 ± 16.45	31.79 ± 10.46	0.741
Parafovea	45.89 ± 8.97	50.79 ± 7.72	0.070
Perifovea	46.38 ± 7.79	44.93 ± 8.12	0.563

*FAZ, foveal avascular zone; BU, Behcet's uveitis; SCP, superficial capillary plexus; DCP, deep capillary plexus. All data are presented as Mean ± SD*.

** *p < 0.01*.

### RPCN Vessel Parameters

The RPCN vessel parameters measured from BU and healthy eyes are summarized in Table 3. The RPCN vascular densities were significantly lower in BU eyes compared to healthy eyes in all sectors (*P* < 0.05) except for the temporal quadrant (*P* = 0.947). Qualitative vascular abnormalities were also observed in the peripapillary region of BU eyes (Figure 2).

**Table 3:** Comparison of Vascular Density in the RPCN between BU and Normal Eyes.

**Variables**	**BU**	**Normal**	***P value***
Peripapillary	48.54 ± 3.66	51.66 ± 5.06	0.005^**^
Superior hemi	48.84 ± 4.49	51.97 ± 5.57	0.004^**^
Inferior hemi	48.15 ± 3.54	51.35 ± 4.92	0.031^*^
Inferior	47.70 ± 5.19	54.12 ± 5.80	0.000^**^
Superior	47.87 ± 6.05	53.12 ± 5.77	0.002^**^
Temporal	53.70 ± 5.04	52.13 ± 8.69	0.947
Nasal	44.91 ± 4.20	48.45 ± 4.03	0.004^**^

*RPCN, radial peripapillary capillary network; BU, Behcet's uveitis. All data are presented as Mean ± SD.*

* *p < 0.05*.

** *p < 0.01*.

### FRT and pRNFLT Parameters

The OCTA findings of FRT and pRNFLT parameters in BU and healthy eyes are summarized in Table 4. The FRT values in the different sectors within the macular region were significantly greater in BU patients than healthy subjects (*P* < 0.05), the more subtly separated analysis of FRT listed in Supplementary Table 2. Similar tendencies were also detected in the pRNFLT, with significantly higher values in all sectors of BU patients compared to controls (*P* < 0.05) with the exception of the nasal quadrant (*P* = 0.075).

**Table 4:** Comparison of FRT and pRNFLT Measurements Evaluated between BU and Normal Eyes.

**Variables**	**BU**	**Normal**	***P value***
**FRT (μm)**			
Whole image	338.25 ± 56.90	290.17 ± 18.46	0.001^**^
Superior-hemi	338.33 ± 58.55	293.67 ± 16.78	0.001^**^
Inferior-hemi	337.83 ± 56.98	286.78 ± 20.17	0.000^**^
Fovea	334.83 ± 155.48	236.17 ± 28.37	0.005^**^
Parafovea	377.04 ± 87.33	322.50 ± 14.50	0.006^**^
Perifovea	338.67 ± 54.64	290.39 ± 20.30	0.000^**^
**pRNFLT (μm)**			
Peripapillary	154.48 ± 62.73	116.63 ± 12.37	0.009^**^
Superior-hemi	152.09 ± 56.19	117.71 ± 12.68	0.010^*^
Inferior-hemi	151.65 ± 70.71	115.42 ± 13.40	0.024^*^
Inferior	189.00 ± 86.73	144.17 ± 19.48	0.034^*^
Superior	183.26 ± 76.36	139.75 ± 17.34	0.013^*^
Temporal	123.09 ± 42.97	82.48 ± 15.84	0.000^**^
Nasal	139.00 ± 88.78	103.67 ± 19.98	0.075

*FRT, full retinal thickness; pRNFLT, peripapillary retinal nerve fiber layer thickness; BU, Behcet's uveitis. All data are presented as Mean ± SD*.

* *p < 0.05*.

** *p < 0.01*.

### Correlations Among Parameters

We first analyzed the correlation between OCTA-based parameters and log MAR BCVA in BU eyes. While there were no correlations between log MAR BCVA and macular parameters (including FAZ area, FAZ perimeter, FRT, choriocapillaris flow area, SCP density, and DCP density), RPCN vascular density was negatively correlated with log MAR BCVA (*r* = −0.692, *p* = 0.013). The pRNFLT showed no significant correlation with BCVA. Pearson correlation coefficients and corresponding *P* values are listed in Table 5, Supplementary Table 3.

**Table 5:** The Results of Pearson Correlation Analyses between log MAR BCVA and Vascular Densities in BU Group.

**Variables**	**Peripapillary**	**Fovea**		**Parafovea**		**Perifovea**	
	**RPCN-VD**	**SCP-VD**	**DCP-VD**	**SCP-VD**	**DCP-VD**	**SCP-VD**	**SCP-VD**
log MAR BCVA	−0.692 (0.013)^*^	0.082 (0.789)	−0.258 (0.394)	0.119 (0.698)	−0.286 (0.344)	0.311 (0.301)	−0.253 (0.405)

*BU, Behcet's uveitis; log MAR, logarithm of the minimum angle resolution; RPCN, radial peripapillary capillary network; BCVA, best-corrected visual acuity; SCP, superficial capillary plexus; DCP, deep capillary plexus; VD, vascular density. All data are presented as Pearson correlation coefficients and their corresponding P values*.

* *p < 0.05*.

The results of correlation analysis between peripapillary and parafoveal vascular densities in BU patients are summarized in Table 6, which reveals a positive association of vascular density between the temporal quadrant of the RPCN and the nasal quadrant of both the parafoveal DCP (*r* = 0.490, *p* = 0.018) and SCP (*r* = 0.309, *p* = 0.023). Across the entire region, however, there were no significant correlations between parafoveal vascular densities (including the vascular density of the SCP and DCP) and peripapillary vascular density. No attractive correlation was located in vascular densities of fovea and perifovea compared to that of peripapillary region (Supplementary Table 4).

**Table 6:** The Results of Pearson Correlation Analyses between RPCN and Parafoveal Vascular Densities in BU Group.

**Variables**		**Parafovea**		**Nasal quadrant of parafovea**	
		**SCP-VD**	**DCP-VD**	**SCP-VD**	**DCP-VD**
RPCN-VD	Peripapillary	0.180 (0.935)	0.251 (0.249)		
	Peri-temporal			0.309 (0.023)^*^	0.490 (0.018)^*^

*BU, Behcet's uveitis; RPCN, radial peripapillary capillary network; SCP, superficial capillary plexus; DCP, deep capillary plexus; VD, vascular density; Peri-temporal, temporal quadrant of peripapillary. All data are presented as Pearson correlation coefficients and their corresponding P values*.

* *p < 0.05*.

Additionally, thicker pRNFLT was correlated with increased FRT in the fovea and parafovea (*r* = 0.534, *P* = 0.009; *r* = 0.454, *P* = 0.029, respectively). In healthy eyes only, there was a positive correlation between RPCN vascular density and pRNFLT (*r* = 0.521, *p* = 0.009). The correlations associated with pRNFLT are summarized in Table 7.

**Table 7:** The Results of Pearson Correlation Analyses between RNFLT and Macular FRT/ RPCN Vascular Density in BU and Normal Eyes.

**Variables**		**FRT-Macular**			**RPCN-VD-PP**
		**Fovea**	**Parafovea**	**Perifovea**	
RNFLT-PP	BU	0.534 (0.009)^**^	0.454 (0.029)^*^	0.148 (0.500)	0.252 (0.246)
	Normal	−0.260 (0.297)	0.311 (0.209)	0.063 (0.804)	0.521 (0.009)^**^

*BU, Behcet's uveitis; RNFLT, retinal nerve fiber layer thickness; PP, peripapillary; FRT, full retinal thickness; RPCN, radial peripapillary capillary network; VD, vascular density. All data are presented as Pearson correlation coefficients and their corresponding P values*.

* *p < 0.05*.

** *p < 0.01*.

## Discussion

BU is a potentially blinding disorder characterized by relapsing-remitting panuveitis with retinal vasculitis involving macular and peripapillary vessels. Not only does it cause extensive vascular leakage or arterial occlusion, but it also triggers severe vitreous opacity (24). As prolonged immunomodulatory therapy in a long-time follow-up is essential for the management of BU (25), the non-invasive OCTA is more suitable for observing microvascular changes compared with invasive FA.

The RPCN cannot be visualized by FA. Alternatively, Engelke and colleagues demonstrated that retinal microvascular changes in the peripapillary region can be evaluated successfully using OCTA (26). Previous studies have identified decreased peripapillary vascular density in glaucoma (14, 27) and non-arteritic anterior ischemic optic neuropathy (28) using OCTA. Therefore, we examined the involvement of peripapillary vasculature in BU using OCTA and found reduced RPCN vascular density in multiple sectors compared to controls, highlighting the potential of OCTA for monitoring disease status among BU patients.

Several studies have conducted quantitative and qualitative assessments of macular zone using OCTA. Vascular abnormalities, including macular capillary hypoperfusion, disorganization, and rarefaction were revealed by qualitative assessment in BU patients (20, 21). Consistent with these reports, macular vascular disorders were also presented in our study cohort (Figure 1). Previous quantitative analyses of the macular vasculature revealed significant reductions in both SCP and DCP densities among BU patients (22, 29). However, the analysis was limited in the parafovea and absent in the perifovea. We found no significant differences in macular vascular densities within parafoveal and perifoveal subfields between groups. Thus, the macular vessel involvement in our BU group was insufficient to be statistically differentiated by OCTA, possibly because our study included patients with retinal edema, which can interfere with vascular quantitative assessment. Significant correlations were found between vascular density in the RPCN temporal sector and vascular densities in the nasal parafoveal DCP and SCP, which could result from the specific nasal position of anatomic feature that the optical nerve head is located in the nasal position of in the macula. In addition, we found a significantly reduced choriocapillaris flow area lower in BU patients compared to healthy controls.

The reasonable unifying explanation for the differences in vascular involvement among regions, is that the extent of damage is associated with the vascular structure and susceptibility to ischemia (even though vasculature damages in BU eyes occur across retinal layers). The RPCN may be more prone to ischemic damage because of the correspondingly dense in unmyelinated nerve fibers with high metabolic demands (9). Besides, the RPCN radiates out of the ONH and branches directly from the central retinal artery and the peripapillary area also contains principal intraretinal vessels compared with macular area (9, 30). Hence, the RPCN vascular density could be more vulnerable to flow changes in larger vessels. Further clinical and histopathological examinations are warranted to clarify the regional evolution of vascular deficits during disease progression and associated functional changes.

When sufficient capillary non-perfusion occurs surrounding the fovea in BU patients, the enlargement and irregularity of foveal avascular zone (FAZ) could be detected by OCTA. A wider FAZ suggests more severe vascular dysfunction, our research demonstrated larger FAZ area and perimeter in BU group compared to controls, although the difference did not reach statistical significance. Likewise, a previous study also demonstrated no significant FAZ changes in BU eyes (31) but significant FAZ differences have been detected (32, 33). The discrepancy in different studies suggests further validation by expanding sample size and that FAZ measurements may not be sufficiently sensitive metrics for determining retinal involvement in BU.

Among anatomic parameters measured, FRT and the pRNFLT were notably greater in BU patients than controls, probably due to retinal edema. We conducted correlation analyses to further clarify the relationship between RNFL thickness and vascular density in the peripapillary region. We found a correlation between RPCN vascular density and pRNFLT in healthy subjects, consistent with previous work (34), but not in BU patients, which may be due to retinal edema and segmentation artifacts.

The main limitation of our study is the relatively small sample size, so these findings require validation, preferably in multi-center studies. A second limitation is the cross-sectional study design, which precluded the assessment of vascular changes during different disease phases. A longitudinal study may reveal the evolution of vascular abnormalities and associations with BCVA deficits.

Nonetheless, our study reveals novel vascular changes in the peripapillary region of the BU retina using OCTA and associations with visual dysfunction. Patients exhibited marked reductions in vascular densities within the peripapillary region, and decreased flow density in the peripapillary zone was mightily associated with poorer BCVA. Taken together, our study indicates that OCTA is a valuable modality for monitoring the microvascular changes associated with BU, and that RPCN vascular density could serve as a sensitive indicator of disease progression and therapeutic response among BU patients.

## Data Availability Statement

The original contributions presented in the study are included in the article/Supplementary Material, further inquiries can be directed to the corresponding authors.

## Ethics Statement

The studies involving human participants were reviewed and approved by the Ethics Committee of Zhongshan Ophthalmic Center (Guangzhou, China 2019KYPJ127). The patients/participants provided their written informed consent to participate in this study.

## Author Contributions

WC, CY, FL, and MH contributed to conception and design of the study. MH, FL, and JL organized the database. CY and XY performed the statistical analysis. CY, FL, and MH wrote the first draft of the manuscript. XY, LS, YH, and WC wrote sections of the manuscript. All authors contributed to manuscript revision, read, and approved the submitted version.

## Funding

This work was supported by National Natural Science Foundation of China (No. 82070950) and Science and Technology Program of Guangzhou of WC (No. 201804010415).

## Conflict of Interest

The authors declare that the research was conducted in the absence of any commercial or financial relationships that could be construed as a potential conflict of interest.

## Publisher's Note

All claims expressed in this article are solely those of the authors and do not necessarily represent those of their affiliated organizations, or those of the publisher, the editors and the reviewers. Any product that may be evaluated in this article, or claim that may be made by its manufacturer, is not guaranteed or endorsed by the publisher.
