# Supplementary material for: Vascular Abnormalities in Peripapillary and Macular Regions of Behcet's Uveitis Patients Evaluated by Optical Coherence Tomography Angiography
Source: Front Med (Lausanne). 2021 Sep 16;8:727151. doi: 10.3389/fmed.2021.727151 (PMC8481620; doi:10.3389/fmed.2021.727151)
Supplement: Supplementary file 1 [file Table_1.DOCX]

Supplementary Material

# Supplementary tables

**Table S1** Comparison of Vascular Densities in Different Sectors of SCP and DCP between BU and Normal Eyes.

| Variables | BU | Normal | P Value |
| --- | --- | --- | --- |
| Vascular density - SCP (%) | | | |
| Parafovea |  |  |  |
| Superior-hemi | 43.43 ± 6.85 | 44.83 ± 6.18 | 0.498 |
| Inferior-hemi | 41.38 ± 7.71 | 43.86 ± 5.69 | 0.258 |
| Temporal | 43.28 ± 7.41 | 45.69 ± 5.52 | 0.254 |
| Superior | 43.78 ± 9.33 | 44.87 ± 6.96 | 0.681 |
| Nasal | 40.40 ± 10.52 | 43.20 ± 6.77 | 0.331 |
| Inferior | 42.15 ± 7.22 | 44.63 ± 5.93 | 0.241 |
| Perifovea |  |  |  |
| Superior-hemi | 48.43 ± 6.55 | 45.53 ± 4.16 | 0.108 |
| Inferior-hemi | 46.98 ± 6.89 | 46.24 ± 4.24 | 0.692 |
| Temporal | 43.21 ± 8.86 | 41.53 ± 5.51 | 0.487 |
| Superior | 48.61 ± 6.48 | 46.08 ± 4.52 | 0.164 |
| Nasal | 52.33 ± 5.19 | 50.20 ± 4.84 | 0.188 |
| Inferior | 48.37 ± 6.16 | 46.97 ± 4.41 | 0.425 |
| Vascular density - DCP (%) | | | |
| Parafovea |  |  |  |
| Superior-hemi | 46.15 ± 10.28 | 51.64 ± 7.59 | 0.064 |
| Inferior-hemi | 45.59 ± 10.30 | 49.95 ± 8.02 | 0.145 |
| Temporal | 46.45 ± 11.83 | 52.22 ± 7.36 | 0.060 |
| Superior | 46.18 ± 11.91 | 49.58 ± 7.67 | 0.298 |
| Nasal | 45.68 ± 11.67 | 52.40 ± 8.93 | 0.048* |
| Inferior | 45.17 ± 11.23 | 48.98 ± 8.64 | 0.238 |
| Perifovea |  |  |  |
| Superior-hemi | 47.55 ± 8.39 | 45.86 ± 8.26 | 0.518 |
| Inferior-hemi | 45.03 ± 8.37 | 43.97 ± 8.19 | 0.683 |
| Temporal | 45.10 ± 11.36 | 47.39 ± 8.05 | 0.473 |
| Superior | 46.85 ± 8.30 | 44.31 ± 8.94 | 0.347 |
| Nasal | 48.81 ± 7.92 | 44.94 ± 9.27 | 0.158 |
| Inferior | 46.16 ± 7.61 | 43.00 ± 8.15 | 0.213 |

SCP, superficial capillary plexus; DCP, deep capillary plexus; BU, Behcet’s uveitis.

All data are presented as Mean ± SD.

*p＜0.05.

**Table S2** Comparison of FRT Measurements Evaluated in Different Sectors between BU and Normal Eyes.

| Variables | BU | Normal | P Value |
| --- | --- | --- | --- |
| Parafovea |  |  |  |
| Superior-hemi | 376.25 ± 86.09 | 323.44 ±14.75 | 0.007** |
| Inferior-hemi | 377.58 ± 90.43 | 321.78 ± 14.53 | 0.007** |
| Temporal | 373.79 ± 90.72 | 313.50 ± 13.15 | 0.004** |
| Superior | 380.17 ± 82.80 | 327.78 ± 13.95 | 0.005** |
| Nasal | 371.38 ± 92.81 | 325.50 ± 20.86 | 0.027* |
| Inferior | 382.83 ± 92.11 | 323.94 ± 13.63 | 0.005** |
| Perifovea |  |  |  |
| Superior-hemi | 340.50 ± 57.09 | 294.28 ± 19.48 | 0.001** |
| Inferior-hemi | 336.42 ± 55.51 | 286.44 ± 21.31 | 0.000** |
| Temporal | 318.35 ± 42.71 | 273.28 ± 17.88 | 0.000** |
| Superior | 340.75 ± 58.42 | 294.39 ± 19.49 | 0.001** |
| Nasal | 354.35 ± 50.89 | 313.39 ± 27.18 | 0.002** |
| Inferior | 339.27 ± 63.24 | 280.50 ± 23.33 | 0.000** |

FRT, full retinal thickness; BU, Behcet’s uveitis.

All data are presented as Mean ± SD

*p＜0.05.

**p＜0.01.

**Table S3** The Results of Pearson Correlation Analysis between OCTA Parameters and log MAR BCVA in BU Group.

| Variables | RNFLT |  | | | FAZ | |  | | FRT | | |  |  |
| --- | --- | --- | --- | --- | --- | --- | --- | --- | --- | --- | --- | --- | --- |
|  | Peripapillary | |  | Area | | Perimeter | |  | Fovea | Parafovea | Perifovea |  | FAC |
| log MAR  BCVA | -0.100 (0.757) | |  | 0.465 (0.176) | | 0.119 (0.210) | |  | 0.119 (0.698) | -0.123 (0.689) | -0.012 (0.968) |  | 0.184 (0.548) |

FAC, Flow area in choriocapillaris;

All data are presented as Pearson correlation coefficients and their corresponding P values.

**Table S4** The Results of Pearson Correlation Analysis between RPCN and Perifoveal/ Foveal Vascular Densities in BU Group.

| Variables | | Foveal region | |  | Perifoveal region | |  | Perifoveal nasal quadrant | |
| --- | --- | --- | --- | --- | --- | --- | --- | --- | --- |
|  |  | SCP-VD | DCP-VD |  | SCP-VD | DCP-VD |  | SCP-VD | DCP-VD |
| RPCN-VD | Peripapillary | -0.154 (0.482) | -0.107 (0.627) |  | 0.055 (0.805) | 0.312 (0.147) |  |  |  |
|  | Peri-temporal |  |  |  |  |  |  | -0.424 (0.149) | 0.125 (0.579) |

All data are presented as Pearson correlation coefficients and their corresponding P values.
